# Stuttering Due To Ischemic Stroke

**DOI:** 10.1155/2005/941926

**Published:** 2005-08-04

**Authors:** Huseyin Alparslan Sahin, Yakup Krespi, Ahmet Yilmaz, Oguzhan Coban

**Affiliations:** ^1^Ondokuz Mayís University Medical School Department of NeurologySamsunTurkey; ^2^Istanbul UniversityInstitute of Neurological SciencesIstanbulTurkey; ^3^Istanbul UniversityIstanbul Faculty of Medicine Department of NeurologyIstanbulTurkey

**Keywords:** stroke, aquired stuttering, supramarginal gyrus, angular gyrus

## Abstract

Acquired stuttering is a disorder of the fluency of speech. The mechanism underlying stuttering is unknown. It may occur after bilateral and unilateral cortical or subcortical brain damage. We report two cases who had stuttering resulting from left parietal infarction.

## 1. Introduction

Stuttering is described as a disturbance in the fluency of speech characterised by involuntary repetitions, blocks, prolongations in producing letters and syllables [8,11]. Although develepmental stuttering is relatively common, affecting about 1% of the population, acquired stuttering secondary to brain damage in adults is considerably rarer [15]. The neuroanatomical and neuropsychological correlates of acquired stuttering are still unknown [8,11]. Acquired stuttering was reported after head trauma, extrapyramidal system disorders, stroke, migraine type headache, and multiple sclerosis [1–4,7,8,12–14].

Here, we present two cases, who had stuttering resulting from left parietal infarction.

## 2. Case 1

A 65 year-old right handed, female with a twenty-year history of hypertension and diabetes mellitus was admitted following the sudden onset of speech disturbance and right-sided weakness. She had no formal education but had learned writing and reading by herself. On neurological examination, she was alert. There was right central facial palsy, and mild paresis and hypoesthesia in the right arm and leg. She had no dysmetria and dysdiadochokinesia. She was able to comprehend, but could not speak. Two days after the onset, her paresis and sensory loss completely disappeared, but she was stuttering. Her motor output was dysfluent characterised by frequent involuntary repetitions, prolongations, and blocks of initial word sounds and syllables. Additionally, secondary stuttering characteristics such as facial grimacing, eye-blinking, fist clenching, and lip tremor were present. There was no adaptation effect. Naming and repetition were normal.

There was no finger agnosia, acalculia, agraphia, alexia, apraxia, right-left disorientation, neglect, and extinction on double simultaneous sensory and visual stimulations. The visual fields were intact on confrontation testing. A computed tomographic scan of the brain showed a small cortical infarct in the left parietal lobe involving Brodmann area 39 and part of Brodmann area 40 (Fig. 1). An echocardiography and Doppler ultrasonography (USG) of the carotid and vertebral arteries were normal. She was treated with antiaggregant, antihypertensive and oral antidiabetic agents. Three weeks after onset, her speech returned to normal.

## 3. Case 2

An 85 year-old right handed male, with 5 year of education presented with a history of hypertension, stable

ISSN 0953-4180/05/$17.00 © 2005 – IOS Press and the authors. All rights reserved

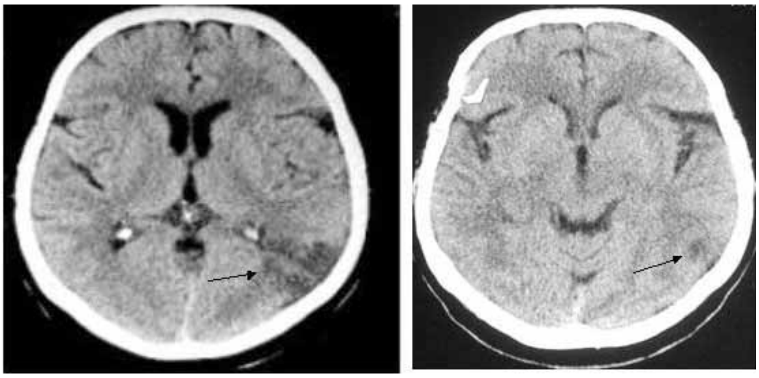

Fig. 1. CT scan of brain showed a cortical infarct on left parietal lobe involving Brodmann area 39 and part of Brodmann area 40.

angina and palpitations. He was admitted because of speech disturbance and right-sided weakness. Thirteen days before admission, he suffered from a transient weakness lasting about 8 hours in the right arm. In the following days, he experienced several attacks lasting about 45 minutes, characterised by right arm weakness and speech disturbance. Two days before admission, the same symptoms became permanent.

On neurological examination, he was alert, and had a mild right hemiparesis-hemihypoesthesia including the face. There was no dysmetria and dysdiadochokinesia. His speech was characterized by repetitions, prolongations and blocks especially on initial syllables and phonemes. Secondary stuttering characteristics and adaptation effect were not present. He had no difficulty understanding written or spoken language. Naming and repetition were normal. There was no finger agnosia, acalculia, agraphia, right-left disorientation, neglect, and extinction on double sensory and visual stimulations. The visual fields were intact by confrontation. Cranial magnetic resonance imaging revealed a cortical infarct in the left posterior parietal lobe including Brodmann area 40 (Fig. 2). There was atrial fibrillation on electrocardiogram and biatrial dilatation on echocardiogram. Doppler ultrasonography of the carotid and vertebral arteries was normal. He was treated with anticoagulant, antihypertensive and antilipidemic agents. When he was discharged from the hospital three weeks after onset, his stuttering had resolved partially. After a few months, he recovered completely.

## 4. Discussion

Both developmental and acquired stuttering are disorders of speech fluency. The pathophysiology of developmental or acquired stuttering still remains an enigma. The clinical features of aquired stuttering are different from developmental stuttering [7,9]. Acquired stuttering has a sudden onset following neurologic insult. Secondary stuttering characteristics such as facial grimacing, eye-blinking and fist clenching are rare in acquired stuttering as compared to developmental stuttering.

Acquired stuttering may occur in association with bilateral or unilateral brain damage. In the reported studies, the lesions resulting in acquired stuttering are located usually in the left hemisphere, but also in the right hemisphere, basal ganglia, thalamus, and pons [1–4, 7,8,11,13,14]. Although the lesion localization is variable, there is no difference in the clinical features of stuttering. Also, the prognosis of patients with small lesions is usually better than that of patients with large lesions.

In our first case, the lesion involved angular (Brodmann area 39) and supramarginal gyrus (Brodmann area 40), but only the supramarginal gyrus (Brodmann area 40) in the second patient in the left hemisphere. Acquired stuttering due to damage of left posterior parietal lobe has been reported previously [14]. For example, Turgut et al., presented a case with stuttering resulting from left posterior parietal infarction, and their patient improved by the first month after onset. The localization of the infarct and the prognosis of our case resemble those of Turgut et al.'s patient.

Angular and supramarginal gyrus are the heteromodal cortical areas which play an important role in language, praxis, visuomotor integrity, motor planning, and spatial attention. They have reciprocal connections with primary cortical, unimodal and heteromodal association areas [6,10]. Whereas the damage of the right angular and supramarginal gyrus causes neglect syndromes and dressing apraxia, the damage of the left leads to finger agnosia, acalculia, agraphia, alexia, ideational/ideomotor apraxia, right-left disorientation,

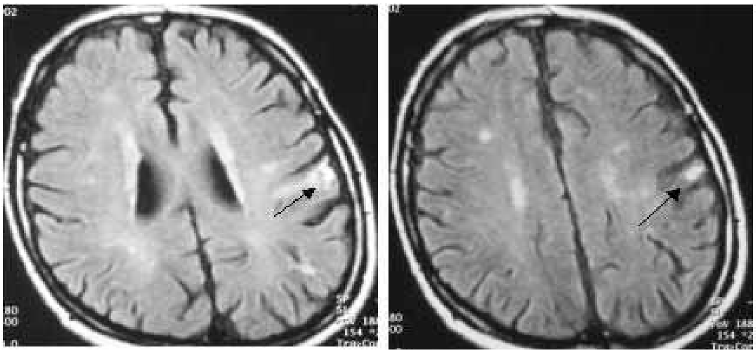

Fig. 2. Cranial magnetic resonance (FLAIR sequence) imaging showing a small cortical infarct on left posterior parietal lobe including Brodmann area 40.

neglect, and extinction [10]. Isolated stuttering is a rare consequence of lesions in this area.

Functional brain imaging studies demonstrated that many cortical and subcortical areas including left posterior parietal cortex are activated while speaking [5,10]. Left supramarginal and angular gyrus play an important role in the motor planning and coordination of speech via inputs to supplementary motor area. Lesions in our cases might have at least partially interrupted the connections of the left supramarginal and angular gyrus to these motor regions and the partial disconnection may have led to the transient episode of stuttering.
